# Compatibility of Different Commercial Alloys in High-Temperature, Supercritical Carbon Dioxide

**DOI:** 10.3390/ma15134456

**Published:** 2022-06-24

**Authors:** Gen Zhang, E Jiang, Weiwei Liu, Hong Yang, Yulong Wu, Yanping Huang

**Affiliations:** 1 Nuclear Power Institute of China, Chengdu 610213, China; ejiang1989@gmail.com (E.J.); cqjiayou2015@126.com (W.L.); zhongguo201908@foxmail.com (H.Y.); huang_yanping09@yeah.net (Y.H.); 2 Institute of Surface Science, Helmholtz-Zentrum Hereon, Max-Planck Str. 1, 21502 Geesthacht, Germany

**Keywords:** supercritical carbon dioxide, oxidation, carburization, high temperature

## Abstract

In this work, the compatibility and long-term integrity of candidate structural materials, including the austenitic stainless steel 316NG, the Fe-Ni-based alloy 800H, and the Ni-based alloy 625, were tested in high-temperature and high-pressure SCO_2_. The exposure time was up to 3000 h. The results showed that the corrosion kinetics approximately followed a near-cubic law for 316NG and 800H. After 3000 h exposure, all oxide layers, mainly composed of Cr_2_O_3_, were continuous, compact, and protective, and their thicknesses were about 21~45 nm, 64~88 nm, and 34~43 nm, respectively. In the case of carburization, dark spots corresponding carbon deposition were observed on the surface and a little enriched in the underside of the oxide for 800H. Moreover, the enrichment of trace elements was found at the oxide/substrate interface through GDOES and TEM analyses, i.e., the enrichment of Mn and Si for 316NG, the enrichment of Mn, Si, Al, and Ti for 800H, and the enrichment of Ti and Al for alloy 625.

## 1. Introduction

Supercritical carbon dioxide (SCO_2_) is considered as one of the prime coolant candidates for next-generation power plants, as it has advantages of higher efficiency, smaller component sizes, fewer components, and a simpler cycle layout, etc. [1,2]. In the SCO_2_ Brayton cycle system, material selection of the key components such as heat exchangers and pipes is one of the important problems of long-term, safe operation [3,4,5,6]. In contrast with the conventional steam Rankine cycle, the prime considerations in regard to material corrosion are that (i) the oxidation of some components at higher temperatures (RT~850 °C) and higher pressures (0.1~30 MPa), and (ii) carburization resulting in material degradation, which is unique to CO_2_ atmospheres.

Over the past several decades, corrosion data regarding SCO_2_ have been gradually accumulated; the current investigations focus on a number of aspects, the first of which is the compatibility of the structural materials [7,8]. Chen et al. [7] reported corrosion and carburization behaviors of austenitic steels (347H and 316LN) and ferritic/martenitic (F/M) steels (430 and 630) with similar chromium content (~17.5 wt.%) and found that spallation of the chromia layer occurred for austenitic steels, which was attributed to the lower diffusion coefficients of Cr, larger growth stresses, and thermal stresses. Moreover, Olivares et al. [8] compared the corrosion resistances of several high-nickel alloys in the air and SCO_2_. Except for low Cr alloy 282, rates of scaling and internal oxidation were, in most cases, similar for all alloys in both gases. According to the published studies, it is clear that the formation of a continuous chromia (Cr_2_O_3_) layer on the substrate materials plays a key effect on their corrosion resistance. In addition to conventional commercial alloys, due to better oxidation and carburization resistance of Al_2_O_3_ scales than those of Cr_2_O_3_, alumina-forming austenitic (AFA) steels [9,10] were also developed to test in high-temperature SCO_2_. Secondly, the effects of temperature and pressure have been investigated [10,11,12,13]. As suggested by Mahaffey et al. [10], weight gain usually increased as testing temperature increased. In contrast, the effects of pressure are relatively complicated. Lee et al. [11] suggested that the weight gain and the thickness of an amorphous Clayer at the oxide/matrix interface increased with increasing the CO_2_ pressure from 0.1 MPa to 20 MPa. However, for 800HT, the depth of the region carburized by Cr-rich carbides was rarely affected by CO_2_ pressure, resulting in similar tensile properties. Thirdly, the effects of impurities in CO_2_, including O_2_, CO, H_2_O, H_2_, SO_2,_ and hydrocarbons, have been investigated [14]. Mahaffey et al. [15] found that oxidation accelerated, resulting in formation of a thicker oxide layer and evidence of spallation, even with the addition 10 ppm O_2_ for the Ni-based alloy 230. Moreover, Oleksak et al. [16] further suggested that O_2_, H_2_O, and SO_2_ had negligible effects on the oxidation of ferritic Fe alloys, while SO_2_ could significantly enhance the rate of oxidation for austenitic Fe alloys. Fourthly, studies have examined the integrity of welding joints, including examinations of the diffusion-bonded technique [17] towards the printed circuit heat exchanger (PCHE) [1,18], the fusion-weld technique [2], and the typical gas tungsten arc welding (GTAW) technique [19]. Fifthly, studies have examined the preparation of the coating on the metal substrate, such as the deposition of a Cu coating [20], a Si coating [21], an Al coating [22,23], a Cr coating [24,25], and a NiAl coating [26]. Finally, the research has examined corrosion under stress, including static and dynamic stress [27,28,29], and creep and stress corrosion cracking (SCC), so far.

The progress made through existing research was simply summarized above. The first aspect, the compatibility of the structural materials, is the foundation for all other studies. However, there are also contradictions between different studies. For example, Chen et al. [7] reported that spallation of the chromia layer occurred for austenitic steels, but not for F/M steels. However, because thermal expansion is similar for both F/M steels and its scales, some researchers found that, although a thick oxide layer formed on F/M steels, its adherence to the substrate was better than that of austenitic steels [28]. Moreover, there are also contrary results on whether an amorphous C layer forms at the oxide/substrate interface, or if Cr-rich carbides form in the substrate. This may because the exposure time used in some experiments was only 1000 hours, or even only 100s of hours. There doesn’t seem to be any further need to predict the corrosion lifetime of structural materials. This work aims to systematically investigate the corrosion and carburization behaviors of austenitic stainless steel (316NG), an Fe-Ni based alloy (800H), and a Ni-based alloy (625), through considerations including (i) a corrosion kinetics curve during 3000 h of exposure, (ii) surface morphology and composition of the scale using SEM and XRD/Raman analysis, (iii) elemental distribution of a cross section of the scale using GDOES analysis, and (iv) elemental enrichment at the oxide/substrate interface including the structure, thickness, and phase component of the scale, using TEM analysis.

## 2. Experimental Methods

### 2.1. Materials and Specimen Preparation

**Figure 1:**
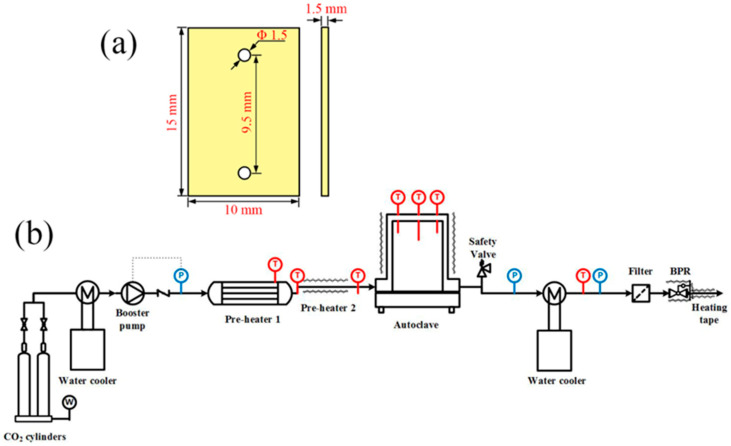
(**a**) Geometry and dimensions of coupon type specimens and (**b**) schematic of the SCO_2_ compatibility testing system.

The alloys used for the exposures were acquired from commercial manufacturers, and the chemical compositions, detected by inductively coupled plasma mass spectrometry, are provided in Table 1. Prior to the experiment, the alloys were cut to coupon type specimens following standard JB/T 6074-92, and the detailed dimensions are shown as Figure 1a. The coupon-type specimens were then ground on all surfaces up to 2000 grit using SiC papers, followed by being dried in warm air.

Figure 1b shows a schematic of the SCO_2_ compatibility testing set-up used in this study, comprised by the following five components: (i) a CO_2_ supply system in which CO_2_ cylinders with 99.99% research grade were used for this study. From the cylinders, the CO_2_ gas traveled through the tubing and flowed into (ii) a liquid booster pump, which could increase the pressure to the desired value and bring CO_2_ to the test section. To make sure the CO_2_ flowing from the cylinder to the booster pump was in a liquid state, a water cooler was used to prevent the CO_2_ gas from gasifying through the tubing. The liquid CO_2_ was then heated up to 500 °C through (iv) a pre-heater and became the supercritical fluid. The SCO_2_ then flowed into (v) the autoclave (test section) which contained the test specimens. The pressure of the autoclave was maintained at 20 MPa by a back pressure regulator (BPR) and the temperature of the autoclave was controlled within ±2 °C by three zone main heaters. Eventually, the SCO_2_ flowing from the BPR was cooled and its pressure was released. The BPR and the tubing following the BPR were wrapped in Omega lux heating tape, which prevented CO_2_ from freezing to plug the pressure-relief vent.

At the beginning of test, twelve duplicate coupons of each alloy were mounted on an alumina sample train and were then put into the autoclave. In this study, the exposing time was up to 3000 h. After 100 h, 200 h, 500 h, 1000 h, 2000 h, and 3000 h exposure to SCO_2_, the autoclave was cooled to room temperature and the pressure was decreased to the atmospheric pressure. Subsequently, all of specimens were removed from the autoclave, photo-documented, and weighed. Two specimens of each alloy were reserved for further analysis, while the others were returned to the autoclave for further testing.

### 2.2. Characterization

After each test, the weight gain of the specimens was measured using a microbalance with a resolution of 0.01 mg. Scanning electron microscopy (SEM; FEI nova 450, Pleasanton, CA, USA) equipped with an EDS detector was employed to investigate the surface morphology as well as an elemental analysis of the oxide layers formed during exposure process in SCO_2_. X-ray diffraction (XRD; Panalytical XPert, Amsterdam, Holland) was applied to study the crystal structure and phase composition of the oxide, using a Cu Kα radiation (25 kV, 40 mA) at a glancing angle of 1.5°, in the range of 2θ from 10 to 80°. Raman spectra were obtained with an argon laser (Renishaw 2000, Oxford, UK). Depth profile analysis was carried out using glow discharge optical emission spectroscopy (GDOES; GDA750HP, Berlin, Germany) with an anode 4 mm in diameter. A transmission electron microscope (TEM; FEI Themis Z, Pleasanton, CA, USA), equipped with a high-angle angular-dark-field (HAADF) detector and an EDS system, was used at 300 kV for electron diffraction, high-resolution scanning transmission electron microscopy (STEM) imaging, and composition analysis. A focused ion beam (FIB; FEI Helios G4, CX, Pleasanton, CA, USA) was used to prepare the TEM specimens, through the lift-out method. The detailed preparation process of the FIB lamellas was divided into four parts: (1) the ion-beam deposition of Pt layer with 1 µm; (2) a U-cut from samples using a Ga ion beam; (3) the weld lamella with an omniprobe, and life out; (4) the deposition of Pt to attach lamella with a Cu grid; (4) the thinning down to a thickness of 50 nm.

## 3. Results and Discussion

### 3.1. Weight Gain and Morphology

**Figure 2:**
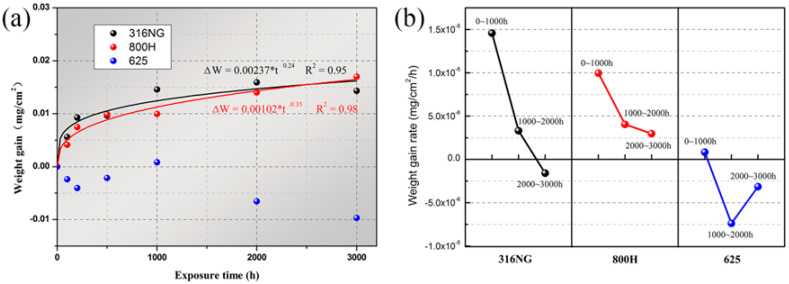
(**a**) Weight gains and (**b**) oxidation rate during 3000 h exposure in SCO_2_ for 316NG, 800H, and alloy 625.

The weight gains of 316NG, 800H, and 625 alloys during 3000 h exposure to SCO_2_ are shown in Figure 2a. The weight of both 316NG and 800H quickly increased at first and continuously increased with exposure time, while that of alloy 625 slightly decreased at first (<200 h) and then increased, followed another decrease. It is clear that the corrosion kinetics of 316NG and 800H approximately followed a near-cubic law, which represented a diffusion-controlled oxide growth rate [30]. The fitting lines based on the formula [31,32,33] $\Delta W=kt^{n}$ are also given in Figure 2a. The lower value of the exponent (0.24 for 316NG and 0.35 for 800H) indicated a slow oxide growth kinetic for both alloys. In contrast, the kinetic process of alloy 625 was relatively complicated. Figure 2b shows the oxidation rate for each alloy at every 1000 h of exposure. For 316NG and 800H, the oxidation rate significantly decreased with exposure time, which indicated the formed oxide film was protective towards the underlying alloy substrate. However, at the initial exposure stage (0~1000 h), the oxidation rate of alloy 625, with its high Cr and Ni content, was 1~2 orders of magnitude lower than that of 316NG and 800H.

**Figure 3:**
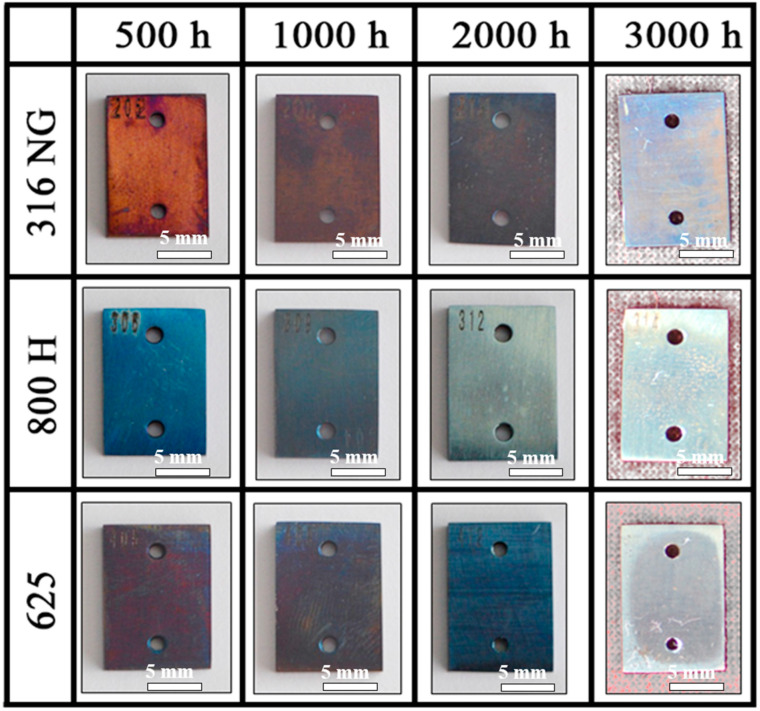
Photographs of 316NG, 800H, and 625 alloys after exposure for 500 h, 1000 h, 2000 h, and 3000 h in SCO_2_.

Figure 3 shows photographs of 316NG, 800H, and 625 alloys after exposure for 500 h, 1000 h, 2000 h, and 3000 h. In the case of the evolution of their surface colorations, a significant difference was observed. After 500 h exposure, the surface of 316NG had a rusty red tint, which might be attributed to the oxide of Fe. This is because the content of Fe in 316NG is highest among these three kinds of steels (see in Table 1). However, after 3000 h exposure, the surface color of 316NG turned to light blue, suggesting that a transformation of the oxide film has taken place (proved by the Raman and GDOES analysis). As for 800H, the color of its surface changed from dark blue to grey, and the color of alloy 625 changed from dark brown to dark blue then, finally, to grey. The composition and state of the oxide film formed on steels determined the color. The transformation of the surface oxide film was detected by the Raman and GDOES analyses. It has often been suggested that the evolution of a thicker oxide layer, possibly of different stoichiometries, and the carbon deposition on the surface result in the change in color during exposure [30,34]. In addition to the change of color, no obvious breakaway oxidation or cracking was observed for any of the specimens.

### 3.2. SEM Surface Morphology

**Figure 4:**
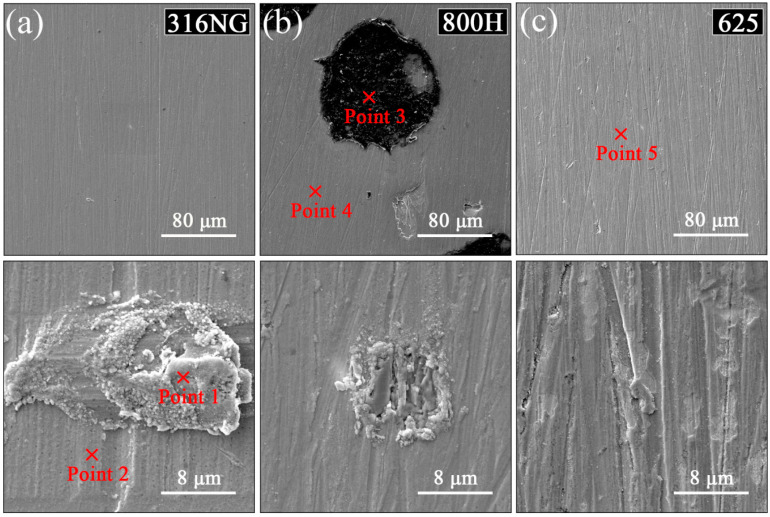
Surface morphologies of (**a**) 316NG, (**b**) 800H, and (**c**) 625 alloys after 3000 h exposure in SCO_2_. The chemical compositions of point 1, 2 (316NG), point 3, 4 (800H) and point 5 (625) were analyzed by EDS.

Figure 4 shows the surface morphology of 316NG, 800H, and 625 alloys after 3000 h exposure, and the corresponding EDS quantifications are listed in Table 2. Except for 800H, there was no obvious change in the surface morphology up to 3000 h exposure, and the initial grounding traces were visible for both 316NG and alloy 625. Some dark spots were randomly distributed on the surface of 800H. The EDS compositional analysis (shown in Table 2) indicated that these dark spots were a result of carbon deposition from the CO_2_-containing atmosphere. Moreover, at a higher magnification, corrosion products occurred at a local defect position for 316NG and 800H. EDS analysis results indicated that carbon was detected on the surfaces of all three specimens. It is also worth noting that the true composition of oxides may not agree with that determined by EDS; this may be because the EDS detector picked up signals from a few microns beneath the specimen surface.

### 3.3. XRD and Raman Analysis

**Figure 5:**
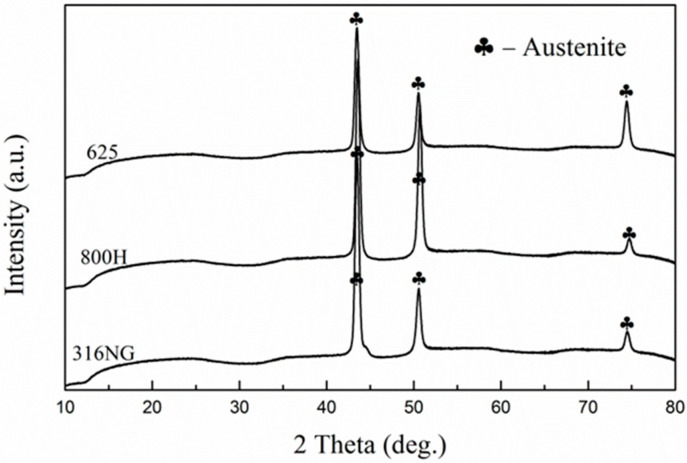
XRD pattern of 316NG, 800H, and 625 alloys after 3000 h exposure in SCO_2_.

**Figure 6:**
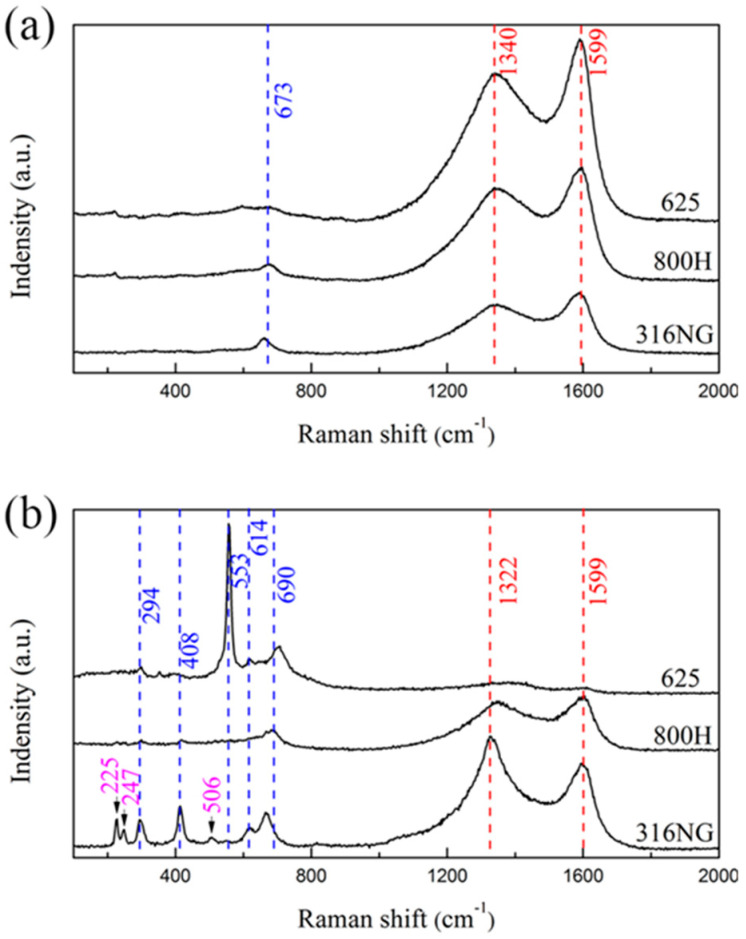
Raman results of 316NG, 800H, and 625 alloys after (**a**) 1000 h and (**b**) 3000 h exposure in SCO_2_.

Figure 5 reveals the XRD pattern of 316NG, 800H, and 625 alloys after 3000 h exposure. Except for the diffraction peaks related with γ-Fe, no obvious diffraction peak was found, as shown in Figure 5. This result indicated that the formed oxide layer was so thin that the composition and structure could not obtained through XRD measurement, even with a low incidence angle of diffraction. Furthermore, Raman measurements were performed after 1000 h and 3000 h exposure, and the obtained results are shown in Figure 6. As shown in Raman spectra, a weak peak was detected after 1000 h exposure, corresponding to Cr_2_O_3_. The main peaks, at 1340 and 1599 cm^−1^, were also due to carbon deposition on the surface [3,4,15]. The above results confirmed that the continuous oxide film might be too thin to detect through Raman analysis after 1000 h exposure in the 500 °C and 20 MPa SCO_2_ environment. Continuing to prolong the exposure time to 3000 h, the oxide films of 800H and alloy 625 were consisted by Cr_2_O_3_ (Raman shift marked with blue). For 316NG, Raman peaks correlated with where Fe_2_O_3_ was detected as well, which may be due to the material’s high Fe content (~67 wt.%), compared with the other two.

### 3.4. GDOES Analysis

**Figure 7:**
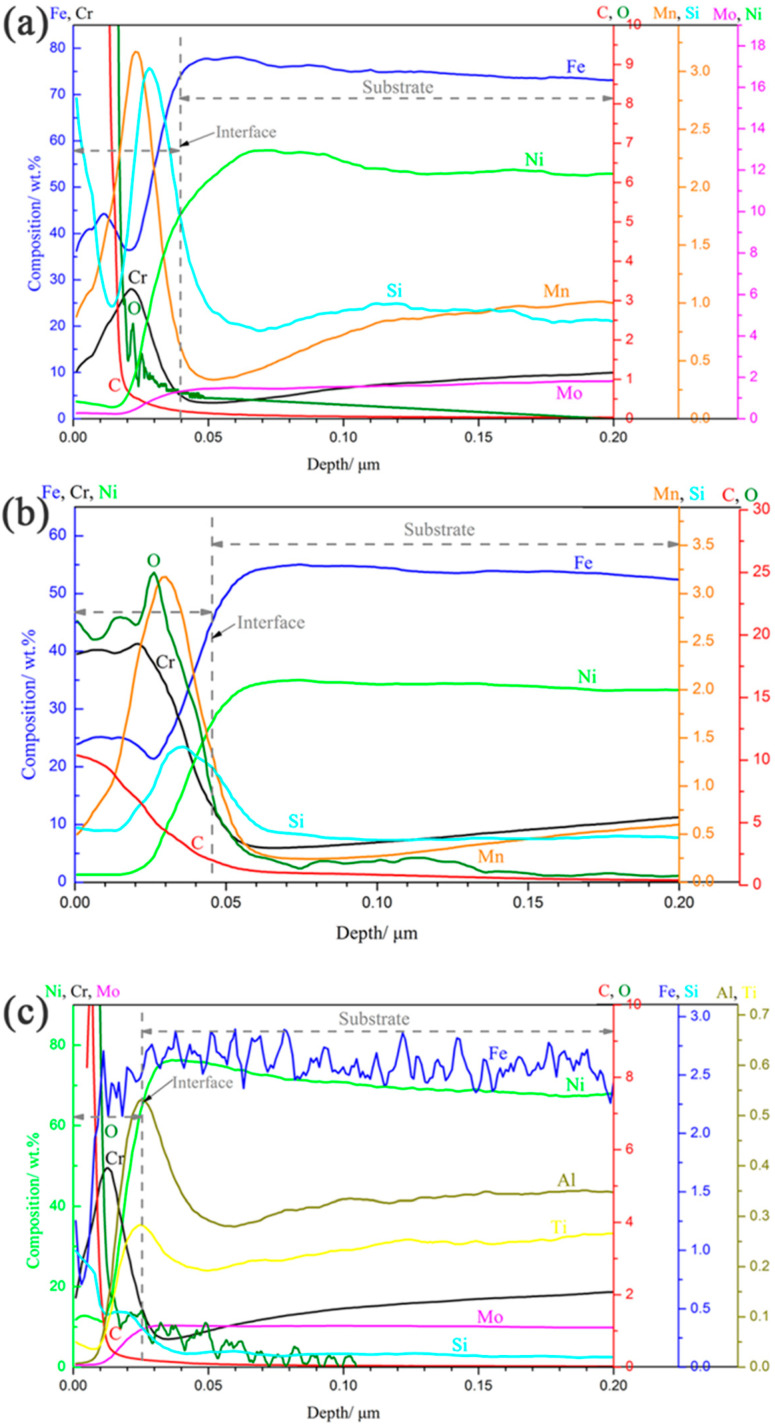
GDOES results of (**a**) 316NG, (**b**) 800H, and (**c**) 625 alloys after 1000 h exposure in SCO_2_.

The elemental distribution of a cross section of the oxide layer after 1000 h exposure was analyzed using GDOES, and the obtained result is shown in Figure 7. For all the specimens, a Cr-depletion zone formed at the interface between the oxide layer and the substrate, which was due to the outward diffusion of Cr from the substrate and formation of a Cr-rich oxide at the gas/solid interface. Furthermore, Mn and Si for 316NG, Mn and Si for 800H, and Al and Ti for alloy 625 enriched at the respective substrate/oxide interfaces. Researchers suggested that Mn from the underlying substrate may be partially consumed to form the (Cr, Mn)_3_O_4_ or Mn_1.5_Cr_1.5_O_4_ spinel oxides near the substrate/oxide interface [7,11]. A very thin and amorphous Si-rich oxide layer was also found in the interface in a study examining 630 SS and 430 SS, which seems to support our results. It was reported that an internal silica layer could limit diffusing cations and anions, and this was attributed to the fact that its structure could aid the formation of the external chromia layer [35]. In addition, as suggested by Lee [5] and Tan [33], Al_2_O_3_ and TiO_2_ could form at the interface between a chromia layer and the substrate as they are stable at a much lower oxygen partial pressure. In case of carburization, the enrichment of C occured not only at the air/solid interface, but also underneath the oxide for 800H. However, for 316NG and 625 alloys, the enrichment of C occurred mainly on their surfaces. Finally, the approximate thicknesses of the oxide layers after 1000 h exposure could also be obtained from the GDOES results, and the values were about 40 nm, 45 nm, and 25 nm for the 800H, 316NG, and 625 alloys, respectively.

### 3.5. TEM Analysis

**Figure 8:**
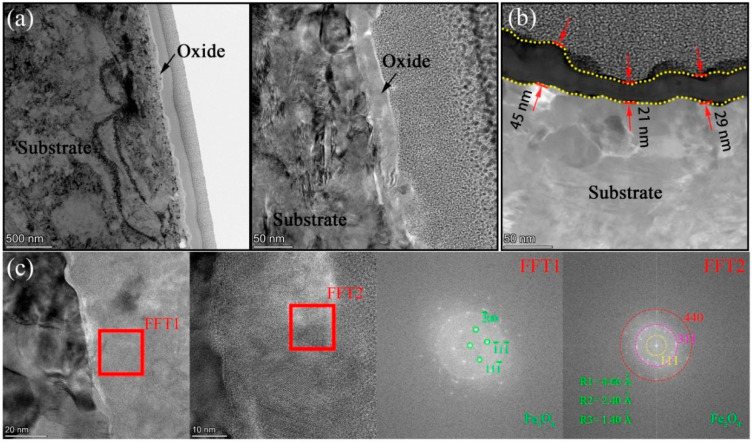
(**a**) STEM micrographs, (**b**) HAADF micrograph, and (**c**) high-resolution STEM and corresponding Fast Fourier Transformation (FFT) micrographs of the cross section of 316NG.

**Figure 9:**
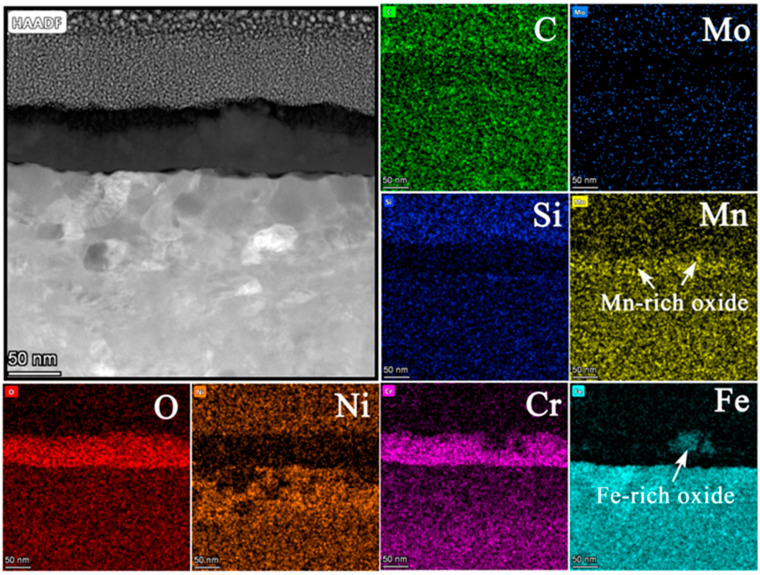
EDS mapping images of the oxide layer of 316NG after 3000 h exposure in SCO_2_.

**Figure 10:**
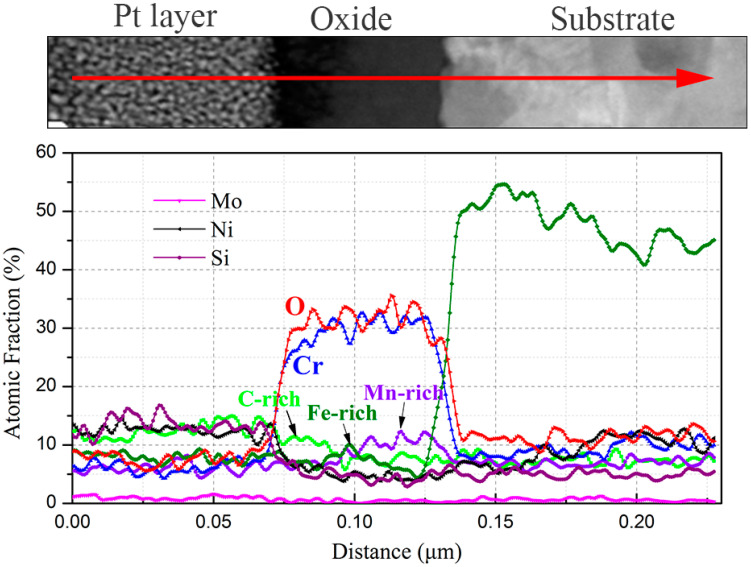
EDS line scanning of the oxide layer of 316NG after 3000 h exposure in SCO_2_.

**Figure 11:**
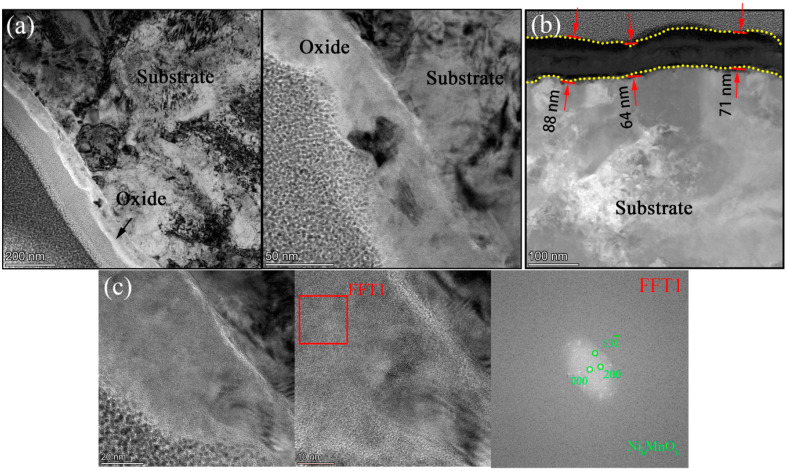
(**a**) STEM micrographs, (**b**) HAADF micrograph, and (**c**) high-resolution STEM and corresponding Fast Fourier Transformation (FFT) micrographs of the cross section of 800H.

**Figure 12:**
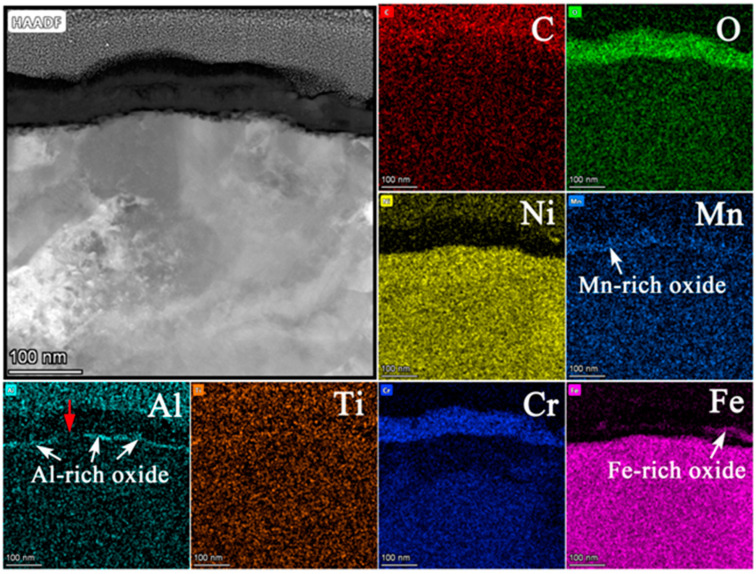
EDS mapping images of oxide layer of 800H after 3000 h exposure in SCO_2_.

**Figure 13:**
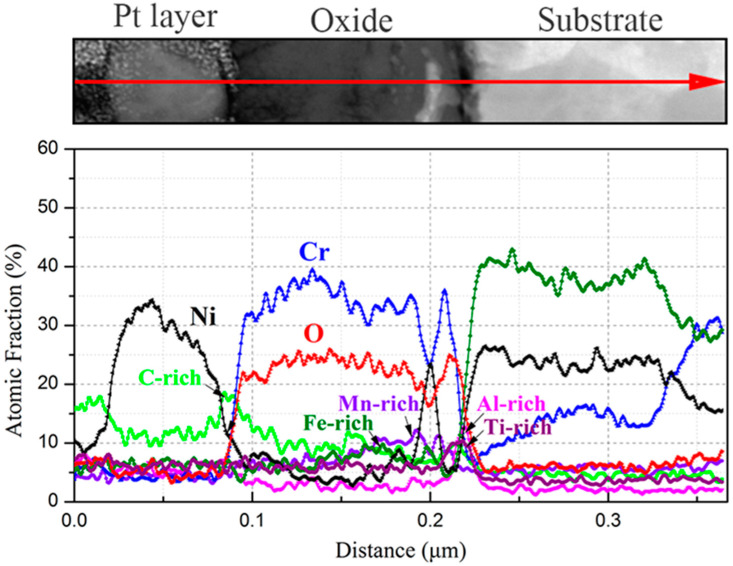
EDS line scanning of the oxide layer of 800H after 3000 h exposure in SCO_2_.

**Figure 14:**
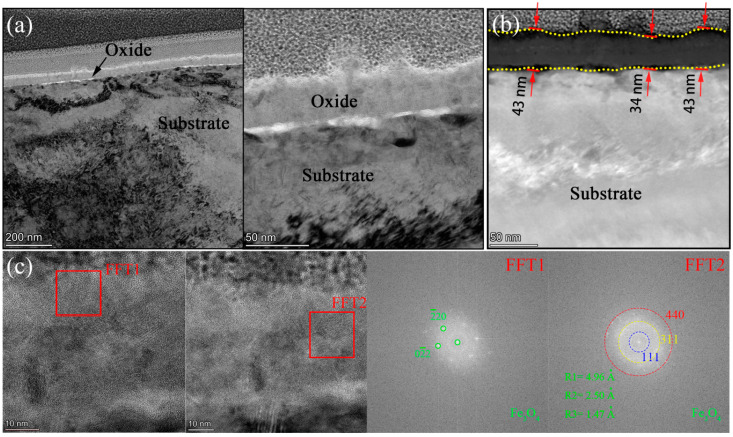
(**a**) STEM micrographs, (**b**) HAADF micrograph, and (**c**) high-resolution STEM and corresponding Fast Fourier Transformation (FFT) micrographs of the cross section of alloy 625.

**Figure 15:**
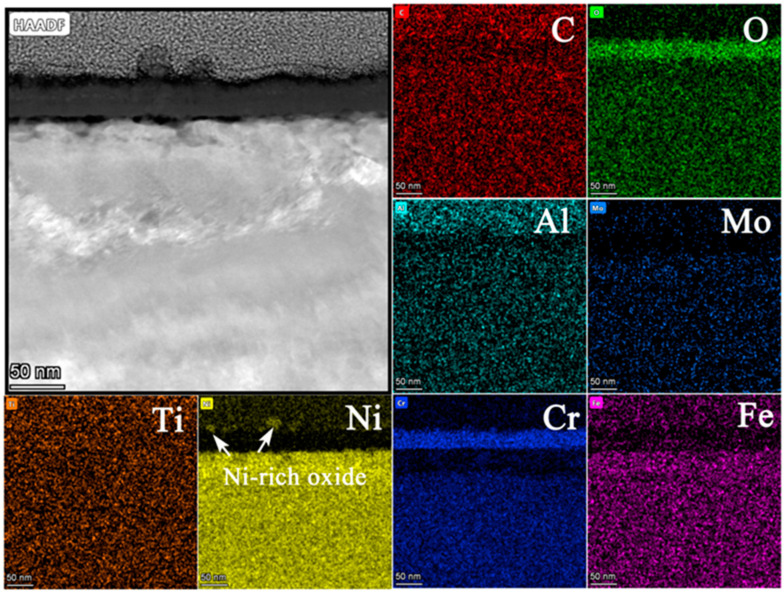
EDS mapping images of oxide layer of alloy 625 after 3000 h exposure in SCO_2_.

**Figure 16:**
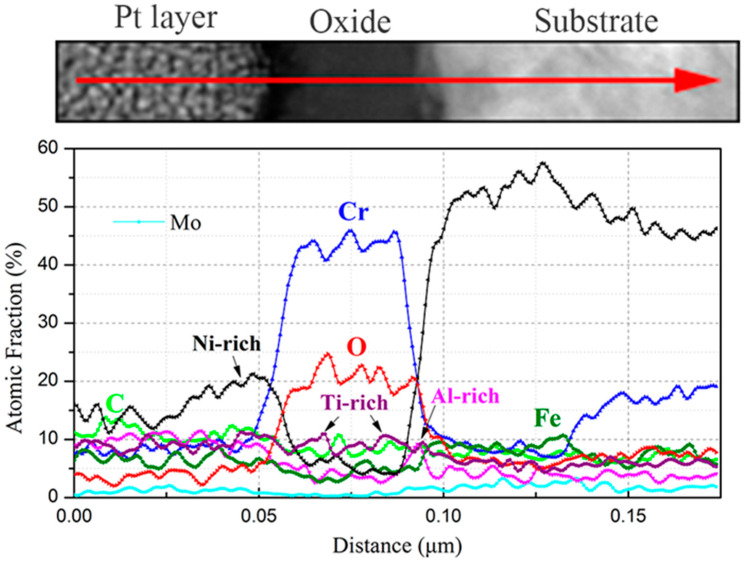
EDS line scanning of oxide layer of alloy 625 after 3000 h exposure in SCO_2_.

In order to uncover the detailed microstructures and chemical composition at the substrate/oxide interface after 3000 h exposure, FIB lamellas containing the oxide layer were lifted out from the 316NG, 800H, and 625 specimens; the TEM/EDS results are shown in Figure 8, Figure 9, Figure 10, Figure 11, Figure 12, Figure 13, Figure 14, Figure 15 and Figure 16. Figure 8 shows (a) the STEM micrographs, (b) the HAADF micrograph, and (c) the high-resolution STEM and corresponding Fast Fourier Transformation (FFT) micrographs of the cross section of 316NG. As shown in the STEM micrographs with lower and higher magnifications, the oxide layer was continuous and compact, but its thickness was not uniform. According to the HAADF micrograph, its average thickness was between 21 nm and 45 nm. Compared with the thickness of the oxide after 1000 h exposure (~40 nm), there was almost no change in thickness, although another 2000 h of exposure was performed. Figure 9 shows the results of EDS mapping near the interface between the oxide layer and the underlying substrate for 316NG. It can be seen that the whole oxide was a Cr-rich oxide, and it was thought that Cr_2_O_3_ combined with the Raman result. Moreover, Mn and Fe accumulated at the interface; in particular, the enrichment of Fe is very clear in Figure 9. Further EDS line scanning (Figure 10) conformed a multilayer structure of the oxide layer of 316NG after 3000 h exposure, i.e., the outer layer of the oxide was C-rich, the middle layer was Fe-rich, and the inner layer was Mn-rich. High-resolution STEM and its FFT micrographs (Figure 8c) showed that the Fe-rich oxide existed in the form of Fe_3_O_4_ or Fe_2_O_3_ (see Figure 6), and no Mn-containing crystal structure was found according to the FFT pattern, probably indicating that the Mn-rich oxide was in the form of an amorphous state. The existence of C in the oxide was due to carburization from the CO_2_-containing atmosphere.

Figure 11 shows (a) STEM micrographs, (b)HAADF micrograph, and (c) high-resolution STEM and corresponding Fast Fourier Transformation (FFT) micrographs of the cross section of 800H. We could see clearly from the STEM micrographs that the oxide layer was continuous, compact, and that its thickness was uniform, which was different to 316NG. From the HAADF micrograph, we could observe that the thickness of the oxide was about 64~88 nm after 3000 h exposure. In contrast with the oxide after 1000 h (~45 nm), its thickness increased by 45~96% at different regions after operating for another 2000 h of exposure. Figure 12 shows the results of EDS mapping near the oxide/substrate interface for 800H. In addition to the enrichment with Mn and Fe, Al was also found to enrich at the oxide/substrate interface. Unusually, it has been suggested that Al exists in form of Al_2_O_3_ with either a crystal state or an amorphous state [5]. The Al_2_O_3_ scales have been proven to be particularly protective in limiting carbon or sulfur species and the water vapor aggressive atmosphere [23]. In particular, its carburization resistance was even superior to that of Cr_2_O_3_ [9,23]. However, as suggested by Lee [23], the presence of an Al-rich surface layer alone is not enough to provide sufficient corrosion and carburization resistance, unless pre-oxidation above 900 °C is undertaken to form a more protective α-Al_2_O_3_ on the material’s surface. In our study, a relatively serious carburization still occurred for 800H, as observed in Figure 7, although Al-rich oxides were still formed at the oxide/substrate interface. The reason could be attributed to the facts that: (i) the Al-rich oxide layer was not continuous (marked with a red arrow in the EDS mapping of Al of Figure 12) and (ii) the temperature used was too low to form protective α-Al_2_O_3_. As shown in Figure 11c, only the Ni_6_MnO_8_ crystal phase and the Cr_2_O_3_ phase were found. According to the FFT pattern, no Al-containing crystal structure also demonstrated this speculation. The EDS line scanning of 800H is shown in Figure 13. The Ni-rich matrix can be found not only on the surface of the oxide, but also at the oxide/substrate interface. Chen et al. [7] reported a similar phenomenon to ours, but Ni only precipitated at the oxide/substrate interface in their study. Due to the lower solubility of Ni in the oxides compared to that in the alloys, the oxides could serve as a diffusion barrier, resulting in a precipitate of Ni accumulating at the oxide/substrate interface. However, further experiments are necessary to examine how Ni diffuses to the surface of the oxide.

Figure 14 shows (a) the STEM micrographs, (b) the HAADF micrograph and (c) the high-resolution STEM and corresponding Fast Fourier Transformation (FFT) micrographs of the cross section of alloy 625. The STEM micrographs indicated that the oxide layer was continuous, compact, and that its thickness was also uniform. From the HAADF micrograph, the thickness of the oxide layer was measured to be between 31 nm and 43 nm after 3000 h exposure. Compared with the thickness of the oxide layer after 1000 h exposure (~25 nm), the thickness increased by 24~72% at different regions after operating for another 2000 h of exposure. Figure 15 shows the results of EDS mapping near the oxide/substrate interface for alloy 625. Enrichment of Ni, similar to that of 800H, was also observed at the surface of the oxide, which was due to higher content of Ni in the alloy substrate (>30%). From the EDS line scanning (Figure 16), it can be observed that Ti enriched at the inside of the oxide and Al enriched at the oxide/substrate interface. According to the high-resolution STEM and its FFT pattern, only a crystal structure of Fe_3_O_4_ was found, except for Cr_2_O_3_, indicating that Ti-rich and Al-rich oxides were in the forms of amorphous states [27]. Moreover, there was no obvious carburization for alloy 625, which agreed with the GDOES and Raman results.

### 3.6. Corrosion and Carburization Mechanism

It is generally known that Cr has a higher diffusivity and a higher affinity for oxygen, resulting in priority formation of a Cr_2_O_3_ scale on the surface of its alloys. As the Cr_2_O_3_ scale grew, Cr was removed from the alloy substrate, causing a Cr-depleted zone. From GDOES results after 1000 h exposure, a Cr-depleted zone was observed on all the alloys. In case of Ni, it had a lower oxygen affinity than Fe, resulting in a diffusion rate into Cr_2_O_3_ scale for Fe that was higher than that for Ni. Therefore, Fe oxide was found in the middle of the oxide in Figure 9 and Figure 12 (corresponding to 316NG and 800H), and it didn’t occur in alloy 625, which may be attributed to a lower Fe content (~2.54 wt.%). As for Si and Al, they tended to form beneath or at the substrate/oxide interface. It has been reported that Al is a beneficial element for improving the carburization resistance of an alloy. A large number of studies have begun to focus on the effects of depositing an Al coating on the surface of alloys [22,23,26]. According to their results, the protective effect of an α-Al_2_O_3_ layer was better than that of an amorphous Al-rich layer alone. No enrichment of Si was observed in our TEM results, which may be because the underlying silica oxide layer was slow to develop, compared to alumina [32]. Mn and Ti are thought to be detrimental to oxidation resistance. They could rapidly diffuse through the chromia scale and form oxide on the outer surface which thickens with time, increasing the overall oxidation rate [35].

As suggested by Lee et al. [5], the carburization can occur in the unique SCO_2_ atmosphere by (i) supplying C to the oxide/substrate interface through the oxide layer, (ii) diffusing through the metal substrate, and (iii) forming carbides with metal elements. Although the solubility of C in the oxide is virtually zero, CO_2_ from the environment can permeate through nano-channels, pores, cracks, or oxide grain boundaries. When CO_2_ reaches the oxide/substrate interface, it will interact with Cr or Fe according to the following reaction (1) [36]:

*x*M + *y*CO_2_ ↔ M*_x_*O*_y_* + *y*CO (1)

Then, C deposition occurs at the oxide/substrate interface through the Boudouard Reaction (2) during SCO_2_ exposure [37]:

2CO ↔ C + CO_2_ (2)

In this way, C gradually accumulates at the oxide/substrate interface and eventually develops into an amorphous C layer, along with the growth of the oxide layer. Once the amorphous C layer is formed, it may act as the source of C to the alloy substrate [5]. Therefore, carbides such as Cr_23_C_6_or Cr_7_C_3_ can be formed in the underlying substrate. In addition, at temperature greater than 425 °C, sensitization may occur in the austenitic stainless steel, also causing the formation of Cr carbides at the grain boundary. It is worth noting that sensitization can also occur in nickel-based alloys and ferritic stainless steels, but at higher temperatures [32]. Lee et al. [11] reported that ultimate tensile strength increased and elongation decreased after exposure in SCO_2_ for 800HT. Furthermore, they suggested that Cr-rich M_23_C_6_ carbides in 800HT contributed to an additional loss of ductility [5]. According to the results in our study, no obvious amorphous C layer or carbides formed for all tested alloys. As for 316NG and alloy 625, C deposition only occurred at the surface of the oxide and inside of the oxide, while carburization was also found at oxide/substrate interface of 800H. As in Figure 13, C enriched at the interface between the Ni-rich matrix and the oxide, which may also indicate that the oxide scale had a degree of carburization resistance. Moreover, generally, the lower Cr activity in the Ni-based alloys and lower solubility of Ni in carbides contributed to the carburization resistance of Ni-based alloys [5].

## 4. Conclusions

The compatibility and long-term integrity of candidate structural materials, including the austenitic stainless steel 316NG, Fe-Ni based alloy 800H, and Ni-based alloy 625, were tested in a SCO_2_ environment with a temperature of 500 °C and pressure of 20 MPa. Subsequently, the oxide formed during 3000 h of exposure was systematically investigated, including corrosion kinetics, morphology, composition, and structure. Based on the above tests and analysis, the following conclusions were drawn:

(1) The corrosion kinetics approximately followed a near-cubic law for 316NG and 800H, but not for alloy 625. Meanwhile, the oxidation rate of alloy 625, with high Cr and Ni content, was 1~2 orders of magnitude lower than that of 316NG and 800H for the first 1000 h of exposure.
(2) From the SEM surface morphologies of 316NG and 625 alloy, there was no obvious change up to 3000 h exposure. However, for 800H, some dark spots, corresponding to carbon deposition (with ~60.44 wt % C content), formed on its surface.
(3) The main composition of the oxide scale was Cr2O3 for all tested alloys, therefore, resulting in a Cr-depletion zone formed at the oxide/substrate interface. After 3000 h of exposure, the thicknesses of the oxide scales for 316NG, 800H, and alloy 625 were 21~45 nm, 64~88 nm, and 34~43 nm, respectively. In contrast with observation at 1000 h of exposure, the highest increase in thickness was 800H, with an increase of 45–96%.
(4) Enrichment of trace elements was observed at the oxide/substrate interface, i.e., enrichment of Mn and Si for 316NG, enrichment of Mn, Si, Al, and Ti for 800H, and enrichment of Ti and Al for alloy 625. Futhermore, they existed in the forms of amorphous oxide states.
(5) In case of carburization, for 800H, enrichment of C was not only in the air/solid interface, but also underneath the oxide. However, for the 316NG and 625 alloys, enrichment of C was mainly on their surfaces.

## Figures and Tables

**Table 1:** Chemical compositions of the used commercial alloys (all in wt.%).

Specimens	Cr	Ni	Mn	Si	C	Fe	Others
316NG	17.34	11.79	1.42	0.42	0.025	Bal.	Mo 2.45
800H	19.70	30.63	0.90	0.25	0.06	Bal.	Al 0.22; Ti 0.35
625	21.46	Bal.	0.03	0.07	0.01	2.54	Mo 8.95; Al 0.36; Ti 0.30

**Table 2:** Elemental compositions of different position in Figure 4 (all in wt.%).

Position	Cr	Ni	Mn	Si	C	Fe	O	Mo	Al	Ti
Point 1	13.48	7.86	-	0.66	9.17	53.58	12.19	3.06	-	-
Point 2	15.17	9.58	1.34	-	8.15	54.54	7.87	2.86	-	-
Point 3	-	8.39	-	-	60.44	16.34	5.24	-	-	-
Point 4	18.39	25.85	-	1.84	4.28	40.07	8.76	-	0.52	0.30
Point 5	14.08	50.37	-	0.08	10.53	1.72	13.76	9.21	0.25	-

## Data Availability

The data presented in this study are available on request from the corresponding author.
